# Factors Associated With Falls Among Residents Living in Long‐Term Care Homes in Ontario

**DOI:** 10.1111/opn.70035

**Published:** 2025-06-25

**Authors:** Lori Rietze, Roberta Heale, Robyn Gorham, Abimbola Akomah

**Affiliations:** ^1^ School of Nursing Laurentian University Sudbury Ontario Canada

**Keywords:** accidental falls, long‐term care, nursing, Ontario, risk factors

## Abstract

**Introduction:**

The prevalence of falls in Ontario‐based long‐term care homes is above the provincial benchmark. There is limited research exploring the reason for such a variation. The research question guiding this study was: *What are the risk factors for falls among all residents in Ontario's LTC homes?*

**Methods:**

A retrospective, population‐based study was conducted using Minimum Data Set assessments for all residents of long‐term care in Ontario between April 2019 and March 2020. Binomial logistic regression analysis was used to determine the significance of the relationship of selected variables to falls.

**Results:**

Findings identified a significant relationship between several variables that were not previously found in the existing literature and falls.

**Conclusion:**

This study has important implications for clinicians and researchers globally as they aim to better understand the increased prevalence of falls in older adults living in residential care.

**Implications for Practice:**

Clinicians are encouraged to consider alternatives to high‐risk medications and closely monitor residents on these medications, implement harm reduction strategies for residents with responsive behaviors, and routinely assess residents for bowel incontinence, cognitive decline, or increased care needs.

**Summary**

- What does this research add to existing knowledge in gerontology?
  ○ This research points to a new factor that may have a positive association with falls in the older adult population: bowel incontinence.
  ○ Some new variables were negatively associated with falls, where their presence resulted in less likelihood of falling, such as the use of diuretics, impaired standing imbalance, impaired long‐term memory, reduced range of motion, gastrointestinal disease, osteoporosis, arthritis and cancer.
- What are the implications of this new knowledge for nursing care for and with older adults?
  ○ Nurses should be aware of the association of bowel incontinence with falls to ensure the risk of a fall is addressed for residents exhibiting this factor.
  ○ The findings confirm that falls in LTC are complex and lists of risk factors should not replace expert nursing assessment and knowledge of the resident.
- How could the findings be used to influence practice, education, research, and policy?
  ○ There is a need for future research to confirm these findings and to modify fall prevention programmes and assessment tools to reflect current risk factors in LTC settings.
  ○ The prevalence of falls in Ontario is above the provincial benchmark requiring comprehensive research, education, and fall prevention programs for LTC residents with ongoing evaluation.

## 1 Introduction

Falls often have devastating consequences for older adults. Falls can be traumatic for older adults, with outcomes ranging from physical injury, chronic pain, reduced cognition, loss of independence and death (Botwinicka et al. 2016; James et al. 2020; Padrón‐Monedero et al. 2020; Public Health Agency of Canada 2014). Given this, strategies to prevent falls are vitally important, especially for residents in long‐term care homes (LTC) who are at a high risk for falls.

In Canada, registered nurses work in all areas of the healthcare system, including LTC. Nurses have a critical role in reducing and preventing falls and we know that the incidence of falls in Ontario's LTC homes is higher than expected benchmarked rates but there is limited data on why this trend is occurring (Chinh et al. 2021; Government of Ontario, n.d.). Falls research in LTC in Ontario has been based on small studies, which may not allow for understanding or prioritisation of care and policy development for falls mitigation. Nurses need comprehensive and current evidence to inform their practice and policies to reduce the risk of falls. The purpose of this study was to better understand the risk factors that increase the risk for residents of Ontario's LTC residents. The variables reflect care settings and characteristics of older adults in settings worldwide, and the results have broad implications for the mitigation of falls.

## 2 Background

### 2.1 Overview of Long‐Term Care in Ontario, Canada

In order to provide a context to the setting and data used in this study, we provide a description of the way in which resident health is assessed in Ontario's LTC homes. This is important as it provides a context to the type and quality of health data that is accessible for research purposes.

Ontario LTC facilities provide a home to people who need continuous nursing care, monitoring, and/or assistance with ADLs (Ontario Long‐Term Care Association, n.d.). The Ontario provincial government regulates care services in LTC by way of the *Long‐Term Care Homes Act*, 2007, and Regulation 79/10, 2010 (Government of Ontario, n.d.). The regulation establishes the standards of care necessary for an LTC home to become licensed (Government of Ontario, n.d.) and states that LTC is required to develop and implement programs to promote high‐quality care in four domains: falls prevention and management, skin and wound care, continence care and bowel management and pain management (Government of Ontario, n.d.). Within these domains, LTC residents are assessed after a change in health status, quarterly and annually, using a tool called the Resident Assessment Instrument‐Minimum Data Set (RAI‐MDS) 2.0 (Government of Ontario, n.d.). Data from the RAI‐MDS are used by Health Quality Ontario to monitor and report on the quality of care in all health sectors of the province (Health Quality Ontario, n.d.). The RAI‐MDS tool was developed in the 1980s in the United States to provide a standard uniform assessment in LTC (Poss et al. 2008). This instrument provides ongoing reliable and valid data on LTC residents (Poss et al. 2008; Burrows et al. 2000; Hutchison et al. 2010; Hawes et al. 1995). In terms of falls, data from the RAI‐MDS provides the incidence of falls within the previous 30 and 180 days in LTC in Ontario. Based on historical data from the RAI‐MDS, falls continue to be a concern in LTC settings as seen by elevated prevalence rates (13%) from 2011 to 2021 compared to the provincial benchmark for falls at 9% (HQO 2020).

### 2.2 Falls Risk Factors

There is extensive literature demonstrating that various factors are associated with falls in LTC settings. Some of these factors are the number of years living in LTC, gait and balance alterations, walking aids, poor muscle strength, level of care (i.e., secured unit or specialised dementia unit), ability to perform ADLs, history of falls, age, diseases/health conditions (i.e., orthostatic hypotension, nutritional insufficiency), dizziness, cognitive impairment (i.e., delirium, depression, dementia, impulsiveness and agitation), sensory deficits (i.e., vision and hearing deficits, neuropathy), foot problems, medication and alcohol usage, aggressive behaviours, environmental factors, the use of restraints, urinary incontinence, pain, and a fear of falling (Castaldo et al. 2020; Montero‐Odasso et al. 2022; Toyoda et al. 2022).

Although existing research provides a summary of the state of knowledge about falls in LTC, there are only two studies that explored fall risk in LTC in Ontario (Krueger et al. 2001; Macri et al. 2017). Of these two studies, Krueger et al. (2001) conducted their study more than 20 years ago and Macri et al. (2017) focused on falls for residents receiving antidepressants. As such, there is very limited research aimed at understanding the cause of falls in LTC settings in Ontario using population data to generalise to the larger target population of LTC residents in Ontario. We are hoping that the findings from this study will provide specific and meaningful information about falls in LTC in Ontario to serve as a starting point for further investigation and for tailoring programmes related to falls prevention.

Taken together, we can see that the incidence of falls in Ontario‐based LTC homes is higher than expected benchmarked rates in Canada. We can also see that there are no recent studies that have explored falls risk factors in LTC homes in Ontario using population data. For these reasons, the research question guiding this study was: *What are the risk factors for falls among residents in Ontario's LTC homes?*

## 3 Methods

### 3.1 Methodology

To generate findings from a population‐based data set of Ontario LTC resident fall risk factors, we designed a population‐based, retrospective, cross‐sectional study (Polit and Beck 2017) designed to answer the question: *What are the risk factors for falls among residents in Ontario's LTC homes?* The dataset included RAI‐MDS for all residents in 623 LTC homes in Ontario between April 2019 and March 2020. Ethical approval for this research was obtained from Laurentian University's Research Ethics Board, file number 6017296.

#### 3.1.1 Procedures: Building a Regression Model

In this study, the dependent variable in our regression model was a fall. The RAI‐MDS defines falls as ‘any unintentional change in position where the resident ends up on the floor, ground or other lower level’ (Canadian Institute for Health Information 2012, 162). The RAI‐MDS indicator that served as the dependent variable was *a fall in the previous 30 days*.

Since the purpose of the study was to identify all factors associated with falls in Ontario‐based LTC homes, a large number of explanatory variables were initially included based on similar studies from other jurisdictions (ArcMap 2019). A comparable item from the RAI‐MDS was chosen for each variable that was identified in the literature and used in the analysis. Independent variables included demographic information (number of institutional years, age and gender), medication use, medical conditions (visual impairment, hearing impairment, cognitive impairment and illness/disease), level of activities of daily living, resident behaviours (presence of aggressive behaviour) and regional/seasonal characteristics (Dhargave and Sendhilkumar 2016; Zhang, Ding, et al. 2019; Zhang, Zeng, et al. 2019; Castaldo et al. 2020). Other RAI‐MDS variables had more than two categories that were combined to reflect either the presence of the item or not and their analysis included variables that were either continuous or dichotomous to determine if there was an association with falls if the variable was present to any degree.

To determine any association of a variable with falls, a list of baseline risk factors was created for further review and study. For example, in the RAI MDS, vision was coded with four possible categories ranging from adequate to highly impaired. In our study, we created a dichotomous variable from vision—if it was present or not. In light of this, we analysed the data using binomial logistic regression to demonstrate a relationship between the dependent and independent variables (Joby 2021). The initial model was tested and variables that were not significant were removed. Variables representing < 5% of the residents were treated as outliers and removed because of the impact on the model as a whole (Statistics 2023).

### 3.2 Data Analysis

Descriptive analysis was completed for the total dataset to summarise the characteristics of the sample. Logistic regression analysis of the variables related to falling within the previous 30 days of the RAI‐MDS assessment is presented in (Table 2). The dataset included 372,469 RAI‐MDS assessments for 101,315 residents. In addition, the number and timing of assessments varied from resident to resident. Since the health status of residents can change dramatically between assessments, we analysed each assessment individually to capture all falls.

Two strategies were implemented to prepare the data for the binomial regression analysis. There were missing data denoting sections that were either irrelevant to the resident or were not completed during the assessments. Seven variables were missing data (hearing, vision, arthritis, osteoporosis, arterial heart disease, cancer, and dementia). Missing values were imputed into each incomplete variable using the expectation maximisation method (Sammaknejad et al. 2019), representing 239,792 responses, up to 64.4% of total cases within the seven variables (Madley‐Dowd et al. 2019). The revised variables with imputed values, replaced the original variables in the dataset.

Next, the age variable was centred and scaled. The final model explained 13.6% (Nagelkerke *R* ^2^) of the variance in falls and correctly classified 83% of cases. The Omnibus Tests of Model Coefficients were significant (*p* = 0.00).

## 4 Results

Twenty‐one percent of residents had a recorded fall within the past 3 months. The mean age of residents was 83.6 years, 67% (*n* = 250,636) were female, 44% (*n* = 164,186) had dementia, and 59% (*n* = 218,558) were taking at least one type of medication (Table 1). In this study, 79% of participants had long‐term memory issues, 79% had short‐term memory issues, and 88% had deterioration in their cognitive status within the past 30 days. Our sample of Ontarian LTC residents had similar cognition to that of other Canadian LTC residents (Canadian Institute for Health Information 2018).

**TABLE 1:** Frequency table (*N* = 372,469).

Variable	Frequency, *F*	Frequency in percentage
Female sex	250,636	67
Urban location	325,795	87
Southern location	341,120	92
Short term memory impairment	304,419	78
Long term memory impairment	238,365	43
Cognition impairment	39,249	88
Eating assistance	317,882	85
Personal hygiene assistance	357,685	96
Dressing assistance	356,380	96
Toileting assistance	349,553	94
Assistance with mobility	335,022	90
Sensory impairment	32,485	9
Impaired range of motion		
Neck	86,206	23
Arm	123,831	33
Foot	82,360	22
Leg	123,060	33
Bowel incontinence	112,131	30
Bladder incontinence	47,128	13
Aphasia	35,573	10
Cancer	13,166	4
Gastrointestinal disease	107,653	29
Diabetes mellitus	107,305	29
Arthritis	56,487	15
Osteoporosis	38,657	10
Urinary tract infection	18,476	5
Delirium	157,931	42
Dementia	208,283	56
Depression	125,120	34
Pain	103,288	28
Hypotension	2505	1
Syncope	6081	2
Hip fracture	19,561	5
Diuretic	95,299	26
Antipsychotic	96,748	26
Antidepressants	218,558	59
Hypnotic	12,988	3
Antianxiety	36,290	10
Analgesia	249,862	67

In identifying the risk factors for falls among residents in LTC in Ontario, Table 2 shows the logistic regression analysis results. This table illustrates the modifiable and non‐modifiable risk factors associated with falls in this population‐based study.

**TABLE 2:** Logistic regression predicting the likelihood of a fall within the last 30 days.

Variable	B	SE	Wald	Sig.	Odds ratio	95% CI	
						Lower	Upper
Demographics							
Number of years in institution	0.066	0.002	905.832	< 0.001	1.068	1.064	1.073
Age	0.006	0.000	150.020	< 0.001	1.006	1.005	1.007
Female	0.342	0.010	1196.25	< 0.001	1.408	1.381	1.435
Seasonal/Geography							
Urban location	−0.032	0.014	5.333	0.021	0.969	0.943	0.995
Fall/Winter	0.039	0.009	18.609	< 0.001	1.040	1.022	1.059
Behaviours							
Persistent anger (verbal expressions with self or others)	0.097	0.011	72.133	< 0.001	1.102	1.078	1.127
Physical abuse to others	0.201	0.016	161.974	< 0.001	1.223	1.186	1.262
Verbal abuse to others	−0.005	0.015	0.125	0.724	0.995	0.966	1.024
Wandering behaviour	0.562	0.013	1973.52	< 0.001	1.754	1.711	1.798
Disruptive behaviour	0.022	0.013	2.998	0.083	1.023	0.997	1.049
Resists care	0.038	0.012	11.053	< 0.001	1.039	1.016	1.063
Behaviour symptoms: recent deterioration	0.017	0.016	1.203	0.273	1.017	0.987	1.049
Activities of daily living							
Assistance with dressing	0.255	0.047	28.754	< 0.001	1.290	1.175	1.416
Eating assistance required	−0.205	0.015	192.603	< 0.001	0.814	0.791	0.838
Toileting assistance required	0.327	0.033	97.444	< 0.001	1.387	1.299	1.479
Personal hygiene assistance required	−0.133	0.049	7.330	0.007	0.876	0.796	0.964
Locomotion on unit	0.265	0.021	161.950	< 0.001	1.303	1.251	1.358
Locomotion off unit	−0.003	0.023	0.017	0.896	0.997	0.954	1.042
ADL: recent deterioration	0.552	0.014	1512.547	1	< 0.001	1.736	1.689
Care needs: recent deterioration	0.333	0.016	451.840	< 0.001	1.395	1.353	1.438
Balance							
Balance standing impaired	0.576	0.021	773.091	< 0.001	1.778	1.708	1.852
Balance sitting impaired	−0.249	0.011	539.959	< 0.001	0.779	0.763	0.796
Range of motion							
Neck: loss of range of motion	−0.010	0.012	0.674	0.412	0.990	0.966	1.014
Arm: loss of range of motion	−0.166	0.012	198.638	< 0.001	0.847	0.828	0.867
Leg: loss of range of motion	−0.036	0.013	8.186	0.004	0.965	0.941	0.989
Foot: loss of range of motion	−0.364	0.015	570.524	< 0.001	0.695	0.674	0.716
Incontinence							
Bowel incontinence	0.035	0.012	8.993	0.003	1.036	1.012	1.060
Bladder incontinence	0.272	0.017	262.187	< 0.001	1.313	1.270	1.357
Urinary continence: recent deterioration	0.180	0.013	182.747	< 0.001	1.197	1.166	1.228
Medical conditions							
Dementia	−0.076	0.015	24.794	< 0.001	0.926	0.899	0.955
Arteriosclerotic heart disease	−0.013	0.019	0.428	0.513	0.987	0.951	1.026
Aphasia	−0.099	0.017	35.507	< 0.001	0.905	0.876	0.935
Cancer	−0.051	0.023	4.842	0.028	0.950	0.907	0.994
Gastrointestinal disease	−0.051	0.010	25.013	< 0.001	0.951	0.932	0.970
Arthritis	−0.070	0.014	24.480	< 0.001	0.932	0.907	0.959
Osteoporosis	0.000	0.016	0.001	0.978	1.000	0.969	1.031
Pain symptoms	0.511	0.015	1188.48	< 0.001	1.667	1.619	1.716
Conditions/diseases that make cognitive, ADL, behaviour patterns unstable	0.227	0.010	523.201	< 0.001	1.255	1.231	1.279
Acute episode/flare up of recurrent or chronic problem	0.163	0.013	158.718	< 0.001	1.177	1.147	1.207
Cognitive impairment							
Short‐term memory impairment	0.224	0.018	157.124	< 0.001	1.251	1.208	1.295
Long‐term memory impairment	−0.044	0.013	12.601	< 0.001	0.957	0.933	0.980
Delirium	0.132	0.010	161.739	< 0.001	1.141	1.118	1.165
Decision‐making cognitive skills impairment	0.074	0.021	12.217	< 0.001	1.077	1.033	1.122
Sensory impairment							
Hearing impairment	0.062	0.015	17.127	< 0.001	1.064	1.033	1.095
Vision impairment	−0.023	0.014	2.672	0.102	0.977	0.950	1.005
Medication use							
Number of medications	0.013	0.001	128.249	< 0.001	1.013	1.011	1.016
Analgesia	0.109	0.011	102.013	< 0.001	1.115	1.092	1.139
Antipsychotic	0.135	0.011	159.904	< 0.001	1.144	1.120	1.168
Antianxiety	0.063	0.015	17.083	< 0.001	1.065	1.034	1.098
Diuretic	−0.166	0.011	217.716	< 0.001	0.847	0.828	0.866
Antidepressant	0.193	0.010	367.728	< 0.001	1.213	1.189	1.237
Hypnotic	0.150	0.023	40.811	< 0.001	1.162	1.109	1.216

### 4.1 Modifiable Risk Factors

The following modifiable independent variables were positively associated with a fall: the number of medications taken by a resident (OR = 1.013, 95% CI [1.011, 1.016]) and the use of any of the following medications within the 7 days before the assessment: antipsychotic (OR = 1.144, 95% CI [1.120, 1.168]); antianxiety (OR = 1.065, 95% CI [1.034, 1.098]); antidepressants (OR = 1.213, 95% CI [1.189, 1.237]); hypnotics (OR = 1.162, 95% CI [1.109, 1.216]) and analgesics (OR = 1.115, 95% CI [1.353, 1.438]). In addition, delirium was positively associated with a fall (OR = 1.141, 95% CI [1.118, 1.164]) as was pain (OR = 1.667, 95% CI [1.619, 1.716]) and toileting self (OR = 1.387, 95% CI [1.299, 1.479]) dressing self (OR = 1.290, 95% CI [1.175, 1.416]), locomotion on unit by self (OR = 1.303, 95% CI [1.251, 1.358]). Several responsive behaviours were associated with falls including physical abuse (OR = 1.223, 95% CI [1.186, 1.262]), persistent anger (OR = 1.102, 95% CI [1.078, 1.127]), wandering (OR = 1.754, 95% CI [1.711, 1.798]) and resisting care (OR = 1.039, 95% CI [1.016, 1.063]). Finally, acute episode/flare‐up of a recurrent or chronic problem and unstable behavioural patterns (OR = 1.177 95% CI [1.147, 1.207]).

The following modifiable independent variables was negatively associated with a fall: diuretic use (OR = 0.847, 95% CI [0.828, 0.866]), personal hygiene (OR = 0.876 95% CI [0.708, 1.852]).

### 4.2 Non‐Modifiable Risk Factors

Several non‐modifiable independent variables were positively associated with a fall: age (OR = 1.006, 95% CI [1.005, 1.007]), female sex (OR = 1.408, 95% CI [1.381, 1.435]), longer length of time in the LTC facility (OR = 1.068, 95% CI [1.064, 1.073]), hearing impairment (OR = 1.064, 95% CI [1.033, 1.195]) as well as short‐term memory impairment (OR = 1.251, 95% CI [1.208, 1.295]), decision‐making cognitive skills impairment (OR = 1.088, 95% CI [1.044, 1.134]). In addition were bowel incontinence (OR = 1.033, 95% CI [1.122, 1.122]), recent deterioration in urinary continence (OR = 1.197, 95% CI [1.166, 1.228]), urinary incontinence (OR = 1.313, 95% CI [1.270, 1.357]), impaired standing balance (OR = 1.778, 95% CI [1.708, 1.852]), assistance with on‐unit locomotion (OR = 1.303, 95% CI [1.251, 1.358]), change in ADLs (OR = 1.724, 95% CI [1.676, 1.773]), chronic condition becoming unstable (OR = 1.255, 95% CI [1.231, 1.279]), and experiencing an acute episodic illness (OR = 1.177, 95% CI [1.147, 1.207]).

The following non‐modifiable independent variables were negatively associated with a fall: dementia (OR = 0.926, 95% CI [0.899, 0.955]), arthritis (OR = 0.932, 95% CI [0.907, 0.959]), long‐term memory impairment (OR = 0.957, 95% CI [0.933, 0.980]), loss of range of motion in the arm (OR = 0.847, 95% CI [0.828, 0.867]), and leg (OR = 0.965, 95% CI [0.941, 0.989]) or foot (OR = 0.695, 95% CI [0.674, 0.761]), aphasia (OR = 0.905, 95% CI [0.876, 0.935]), gastrointestinal disease (OR = 0.951, 95% CI [0.932, 1.0970]) personal hygiene (OR = 0.876, 95% CI [0.796, 0.964]), impaired sitting balance (OR = 0.779, 95% CI [0.763, 0.796]), assistance with eating (OR = 0.841, 95% CI [0.791, 0.838]), living in an urban setting (OR = 0.969, 95% CI [0.943, 0.995]).

## 5 Discussion

Twenty‐one percent of residents had a recorded fall within the past 3 months. This is in line with the prevalence of falls found in international literature, namely 12.5% (Izumi et al. 2002) to 66.7% (Neto et al. 2017), but it remains well above the provincial benchmark target of 9% (HQO 2020). Although further studies need to confirm the findings and add to contextual data, the risk of falls in Ontario‐based LTC homes justifies the continued review of falls in this setting.

Findings from this study offer insight into a novel list of risk factors that are, and are not associated with a risk of falls for Ontario LTC residents. Several of the factors associated with falls in this study are in contrast to the findings of other studies. While the use of a large dataset, representing a population of residents in LTC in Ontario provides a solid foundation for the risk factors for falls, the lack of contextual information points to the need to review the findings and possible explanations with caution. Regardless, the findings provide a baseline of risk factors for falls in the LTC population in Ontario and lay a foundation for further research and recommendations for fall prevention.

### 5.1 Non‐Modifiable Risk Factors

In this study, increasing age significantly increased the likelihood of falls. This is confirmed in other studies which uniformly found age to be a risk factor for falls, likely due to the changes that occur with increased age, such as loss of muscle and reduced sensory acuity (Sousa et al. 2016).

Some studies found that men have a higher risk for falls (Lee et al. 2008; Castaldo et al. 2020; Cameron et al. 2018). Other researchers found that being female was a risk factor for falls (Dhargave and Sendhilkumar 2016; Sousa et al. 2016; Zhang, Ding, et al. 2019; Zhang, Zeng, et al. 2019). In our study, being female increased the likelihood of a fall in the last 30 days. It could be recommended that fall risk assessment should be paid equally to both men and women.

There is existing literature that supports that cancer and gastrointestinal diseases increase one's likelihood of falls. Morris and Lewis (2020) and Wildes et al. (2015) found that the risk of falling was higher for older people with cancer while Chou et al. (2023) found that gastrointestinal diseases were associated with increased falls due to gait instability. These study findings are contrary to our results that residents with aphasia, cancer, and gastrointestinal disease had a small, but significant decreased likelihood of a fall within the previous 30 days.

Krueger et al. (2001) and Berk et al. (2019) found that osteoporosis and arthritis increased one's risk for falls by way of decreased bone density, muscle weakness and painful joints (Tada et al. 2021). The findings from this study do not support this relationship, where residents with arthritis had a decreased likelihood of a fall and there was no significant difference for those with osteoporosis.

Research demonstrates a direct relationship between long‐term memory impairment, decision‐making cognitive skills impairment, and falls (Taylor et al. 2014; Chantanachai et al. 2021). This relationship is in contrast with this study where participants with long‐term memory impairment, however, decision‐making cognitive skills impairment and short‐term memory were significantly more likely to have fallen in the previous 30 days.

There is limited agreement on the relationship between urinary incontinence and fall risk. Moon et al. (2021) and Kuhnow et al. (2022) found that urinary incontinence was a fall risk factor, while Lee et al. (2011) and Anderson and Lane (2020) showed that urinary incontinence had an inverse relationship to falls. In our study, we found that residents with urinary incontinence or a recent deterioration in urinary continence had an increased likelihood of a fall within the last 30 days. There are no studies that assess the influence of bowel incontinence on falls risks but in our data, there was an increased likelihood of falls with residents of LTC settings. Bowel incontinence is an under‐recognised risk factor that needs to be monitored to improve the effectiveness of fall prevention strategies.

Literature associating range of motion with falls is scant. Neto et al. (2017) and Zarei et al. (2020) found that limited range of motion was a significant, positive risk factor for falls. This is not consistent with our findings in that participants with a loss of range of motion in the neck, arm, leg and foot were significantly less likely to have fallen. While limited neck range of motion might affect an individual's ability to visually scan their environment to avoid fall hazards and limitations in foot range of motion can affect the ability of residents to quickly react to uneven floors or tripping hazards (Chiacchiero et al. 2010), identifying a resident with limited range of motion may have prompted staff at these LTC setting to provide additional assistance, thereby reducing their likelihood of falls. There are some research findings (Kuhnow et al. 2022) that suggest an inverse relationship between lower leg range of motion and risk of falls. Our study confirmed these findings where participants with loss of range of motion of the leg demonstrated significantly less likelihood of falls. Perhaps this is related to the use of alternative modes of transportation such as walkers, wheelchairs or scooters.

Dokuzlar et al. (2020), Drummond et al. (2020) and Kuhnow et al. (2022) found that as the elderly required more assistance with ADLs, their risk for falls increased. We had similar findings in our study, in that residents needing assistance with ADLs, such as eating and personal hygiene had an increased likelihood of falling.

Imbalance and vertigo (Dhargave and Sendhilkumar 2016), and poor balance and gait (Dokuzlar et al. 2020; Zhang, Ding, et al. 2019; Zhang, Zeng, et al. 2019) are considered to be risk factors for falls, but their studies did not differentiate between sitting or standing imbalance. In our study, residents with impaired standing balance were significantly less likely to have fallen. In a study by Abou and Rice (2022), community‐dwelling wheelchair users were at an increased risk of falls, although sitting balance was not directly assessed in their study either. In our study, residents with impaired sitting balance were significantly less likely to have fallen within the last 30 days. Poor balance while sitting is an under‐recognised risk factor for falls that needs to be assessed.

A resident's locomotion may affect the likelihood of falls. Locomotion describes how the resident moves between locations on and off the unit (i.e., independently or with supervision/assistance). Castaldo et al. (2020) reported that residents who were dependent on staff for locomotion were less likely to fall. Findings from our study are in contrast with the results of Castaldo et al. (2020) in that residents who needed assistance with on‐unit locomotion were more likely to fall within the last 30 days.

### 5.2 Modifiable Risk Factors

There is existing literature showing a positive association between patients with pain who take opioids (Virnes et al. 2022) and delirium (Kalivas et al. 2023) with falls. Our study demonstrated the positive associations between pain, delirium, an acute episode of a recurrent or chronic problem, deterioration of cognitive decision making, ADLs and unstable behaviour patterns, with falls in the past 30 days. There was no data comparable to these findings in the research literature. If delirium, acute episodes of a chronic condition, or pain are recognised by care providers as variables associated with falls, resources can be put in place to address them which, in turn, may reduce the likelihood of a fall.

Agitation has been associated with falls in LTC residents (Fillit et al. 2021). This is in contrast to the findings of Kron et al. (2003) who also studied persistent anger among residents and did not find a significant association with falling. However, the findings of our study show that five responsive behaviours are associated with greater risk of falls including persistent anger, physical abuse, wandering, resisting care and a recent deterioration in behaviour. In fact, wandering is close to two times increased risk for falls and, as a group of behaviours, this represents a notable modifiable risk. This is an important finding as clinicians may implement strategies such as the Behavioural Support Ontario program (Behavioural Support Ontario 2013) to reduce behaviours for patients who are already at a high risk of falling in LTC settings.

Park et al. (2015) conducted a systematic review and found that using sedatives, hypnotics, and antidepressants was associated with an increased risk of falls for adults over 60 years of age. The use of antidepressants, sedatives, and hypnotics in older adults and their effect on fall risk has been well‐researched, with several studies agreeing that the use of antidepressants and selective serotonin reuptake inhibitors were directly associated with falls (Jung et al. 2022; Lin et al. 2021). Findings from our study showed that all these medication categories increased risk of falls.

The direct relationship between fall risk and diuretic use in the elderly is well‐researched (Bai et al. 2023). Berry et al. (2012) purported that residents on diuretics may attempt self‐transfers and ambulation without assistance in a rush to use the bathroom or that diuretics may increase the risk of dehydration and hypotension, which increases fall risk (Hamrick et al. 2020). However, the findings of our study were in contrast to the association in that diuretics increased the likelihood of a fall occurring in the past 30 days for the Ontario LTC resident participants.

### 5.3 New Falls‐Risk Variables

In summary, study findings demonstrate new factors associated with falls. A new variable associated with falls in our Ontario LTC participants was bowel incontinence. Variables that were negatively associated with falls and were new to the literature were the use of diuretics, impaired standing imbalance, impaired long‐term memory, reduced range of motion, gastrointestinal disease, osteoporosis, arthritis and cancer.

## 6 Implications for Research

Future research needs to be aimed at better understanding how to prevent falls by residents in Ontario‐based LTC. For instance, researchers might focus on clinical strategies that could be used to prevent falls in this population, such as when best to time the administration of high‐risk medications or how to promote safety with residents who have poor balance when sitting. Further, this study introduced several new variables associated with increased fall risk including bowel incontinence, poor balance while sitting, need for assistance with locomotion, delirium, and acute episode of a chronic condition. Additional research, such as prospective studies, needs to be conducted to confirm these results with LTC residents in various geographic locations.

## 7 Implications for Education

Healthcare providers at all levels of care need adequate education surrounding risk awareness and fall prevention strategies. Educators are encouraged to include fall assessment tools in their teaching that are comprehensive of the above‐mentioned variables associated with falls in LTC settings. Some practical suggestions for educators are to: begin fall assessment strategies at entry levels of professional education, consider using simulation to practice peer assessment, incorporate real‐life case studies to practice assessments in a group setting, have targeted clinical assignments of fall risk assessments, and show learners how to set a notification to receive an email when new literature is published about fall risk assessments.

## 8 Implications for Policy

Institutional fall prevention policies need to be implemented, such as: regular medication reviews to ensure adequate treatment and to avoid unnecessary polypharmacy, cautious use of medications that increase the risk of falls including antipsychotics, antidepressants, anti‐anxieties, hypnotics, and analgesics. From a health policy perspective, funding for fall prevention programs needs to be continued, as an investment in quality and preventative care.

## 9 Implications for Environment, Climate, Sustainability

The findings from this study have implications for planetary health. We recommend that the factors associated with falls be considered in the design of LTC homes. For instance, considerations should be taken on the most appropriate placement of bathrooms and effective communication strategies between residents and care providers especially for those with bowel incontinence. In doing so, it is our responsibility to ensure that said designs takes into consideration planetary health by maximising conservation and minimising environmental impacts.

## 10 Limitations and Strengths

Contextual and environmental variables could not be assessed because data were limited to the information captured on RAI‐MDS assessment forms. Though the process of collecting data using these forms is vulnerable to errors and reporting bias since it is completed by healthcare staff in each of the LTC homes and the uniformity of the data collection cannot be determined, the RAI‐MDS tool is reliable and valid (Hutchison et al. 2010; Hawes et al. 1995). The odds ratios in the results were not strong; however, most of the findings were confirmed in the existing literature on fall risk factors. A further limitation is that the results refer to risk factors of a fall in the previous 30 days. A recent fall could have led to an alteration in the actual condition of the residents. One strength of the study was that the participant group was a heterogeneous population living in LTC settings in Ontario, so generalisability to other LTC settings in Ontario and Canada is high.

## 11 Conclusions

The findings of this study are robust and wide‐ranging. They serve to fill identified gaps in fall research in Ontario's LTC by better understanding some of the contextual fall risk factors of these residents that might be attributed to the higher prevalence of falls in Ontario LTC homes compared to the national benchmark for falls. Some findings supported existing research; others were contradictory, and there were many new fall risk factors identified.

This study used data provided by the Canadian Institute of Health Information that is collected quarterly and annually from Ontario's LTC residents by healthcare staff. Data from this study supported that many variables had an impact on the likelihood of a fall in Ontario‐based LTC settings. Clinical and research implications associated with these outcomes were discussed. The results of this study may be useful in guiding the efforts of healthcare providers in reducing falls in Ontario's LTC homes.

## Author Contributions

Roberta Heale, Lori Rietze, and Abimbola Akomah were involved in the research design and data collection. Data analysis and interpretation was led by Roberta Heale. Roberta Heale, Lori Rietze, and Robyn Gorham drafted the final article for submission.

## Ethics Statement

This research was approved by Laurentian University Research Ethics Board, file number 6017296.

## Conflicts of Interest

The authors declare no conflicts of interest.

## Data Availability

The data that support the findings of this study are available on request from the Canadian Institute for Health Information. The data are not publicly available due to privacy and/or ethical restrictions.
